# Loong: An Open-Source Platform for Full-Size Universal Humanoid Robot Toward Better Practicality

**DOI:** 10.3390/biomimetics10110745

**Published:** 2025-11-05

**Authors:** Lei Jiang, Heng Zhang, Boyang Xing, Zhenjie Liang, Zeyuan Sun, Jingran Cheng, Song Zhou, Xu Song, Xinyue Li, Hai Zhou, Yongyao Li, Yufei Liu

**Affiliations:** 1 The National and Local Co-Build Humanoid Robotics Innovation Center, Shanghai 201203, China; jianglei@openloong.net (L.J.); xby@openloong.net (B.X.); liangzhenjie@openloong.net (Z.L.); sunzeyuan222@openloong.net (Z.S.); yongyao.li@openloong.net (Y.L.); 2 Humanoid Robot (Shanghai) Co., Ltd., Shanghai 201203, China; zhangheng@openloong.net (H.Z.); chengjingran@openloong.net (J.C.); zhousong@openloong.net (S.Z.); songxu@openloong.net (X.S.); lixinyue@openloong.net (X.L.); zhouhai@openloong.net (H.Z.); 3 Unmanned Vehicle Research Center, China North Vehicle Research Institute, Beijing 100072, China

**Keywords:** full-size humanoid robot, open-source platform, human-like design, multi-level control architecture, modular system integration

## Abstract

In recent years, humanoid robots have made substantial advances in motion control and multimodal interaction. However, full-size humanoid robots face significant technical challenges due to their inherent geometric and physical properties, leading to large inertia of humanoid robots and substantial driving forces. These characteristics result in issues such as limited biomimetic capabilities, low control efficiency, and complex system integration, thereby restricting practical applications of full-size humanoid robots in real-world settings. To address these limitations, this paper incorporates a biomimetic design approach that draws inspiration from biological structures and movement mechanisms to enhance the robot’s human-like movements and overall efficiency. The platform introduced in this paper, Loong, is designed to overcome these challenges, offering a practically viable solution for full-size humanoid robots. The research team has innovatively used highly biomimetic joint designs to enhance Loong’s capacity for human-like movements and developed a multi-level control architecture along with a multi-master high-speed real-time communication mechanism that significantly improves its control efficiency. In addition, Loong incorporates a modular system integration strategy, which offers substantial advantages in mass production and maintenance, which improves its adaptability and practical utility for diverse operational environments. The biomimetic approach not only enhances Loong’s functionality but also enables it to perform better in complex and dynamic environments. To validate Loong’s design performance, extensive experimental tests were performed, which demonstrated the robot’s ability to traverse complex terrains such as 13 cm steps and 20° slopes and its competence in object manipulation and transportation. These innovations provide a new design paradigm for the development of full-size humanoid robots while laying a more compatible foundation for the development of hardware platforms for medium- and small-sized humanoid robots. This work makes a significant contribution to the practical deployment of humanoid robots.

## 1. Introduction

Full-size humanoid robots, serving as one of the ultimate embodiments of embodied intelligence, aim to replicate human form, locomotion, and interaction capabilities, acting as a critical bridge connecting the physical world with digital intelligence [1]. As defined in this paper, their height must exceed 1773 mm (the 90th percentile for adult male height), enabling them to undertake diverse tasks in complex scenarios such as industrial manufacturing, public service, disaster response, and domestic companionship. However, transitioning humanoid robots from laboratory demonstrations to practical applications still faces severe technical bottlenecks, where their inherent size and complexity significantly hinder the realization of such autonomous, adaptive capabilities [2,3].

Currently, the development of full-size humanoid robots is primarily constrained by three core challenges: (1) Insufficient Biomimetic Mobility: Traditional serial chain or simple rotary joint designs struggle to mimic the multi-degree-of-freedom composite motions and inherent compliance of human joints (such as the shoulder, hip, and ankle). This results in rigid robot movements and a lack of replication of efficient, flexible human gaits and fine manipulation capabilities, ultimately limiting their adaptability in unstructured environments [4,5]. (2) Contradiction Between Control System Real-Time Performance and Complexity: Full-size robots typically possess dozens of degrees of freedom, and leg joints require high torque output to support body movement, leading to large system inertia and strong dynamic coupling. Achieving rapid dynamic balance and gait planning under a high center of mass imposes extreme demands on the computational efficiency, communication real-time performance, and multi-task coordination of the control system. Existing solutions often struggle to balance real-time performance with functional complexity [6]. (3) System Integration and Practicalization Dilemma: Integrating high-power-density actuators within the limited body space presents severe thermal management challenges. Simultaneously, the tight coupling of perception, computation, and control units makes the system difficult to maintain and upgrade. A lack of modular design also hinders rapid functional customization for specific tasks and mass production, thereby increasing costs and reducing practicality [7].

Globally, several outstanding research platforms exist, such as Unitree H1, which focuses on highly dynamic motion [8], the all-electric HRP-5P designed for assembly tasks [9], and the hydraulically driven Atlas Gen2 [10], which demonstrates exceptional mobility. Efforts by institutions like Unitree (H1), AIST (HRP-5P), Ubtech (walker S) [11], Tesla Optimus [12], and Boston Dynamics (Atlas Gen2) [10] have contributed valuable solutions to some of these challenges, but each tends to focus on specific issues. None has yet provided a comprehensive solution that fully addresses the aforementioned challenges. To break through these bottlenecks, this paper proposes and develops “Loong”—an open-source, full-size universal humanoid robot platform. The design philosophy of Loong stems from the deep integration of bionic principles and engineering practicality. We not only pursue highly biomimetic joint structures to enhance motion anthropomorphism but are also committed to resolving the real-time control challenges of complex systems through a multi-level control architecture and employing a modular system integration strategy to improve the robot’s maintainability, scalability, and scenario adaptability.

The remainder of this paper is structured as follows: Section 2 will elaborate on the system design of Loong, including biomimetic joints, the multi-level control architecture, and the modular integration strategy. Section 3 validates Loong’s basic performance and scenario application capabilities through a series of experiments. Section 4 concludes the paper and outlines future work. We firmly believe that this open-source platform can provide a new design paradigm for the practicalization of full-size humanoid robots and promote collaborative innovation and standardization within the field.

## 2. Robot Designs

### 2.1. Overview

**Figure 1:**
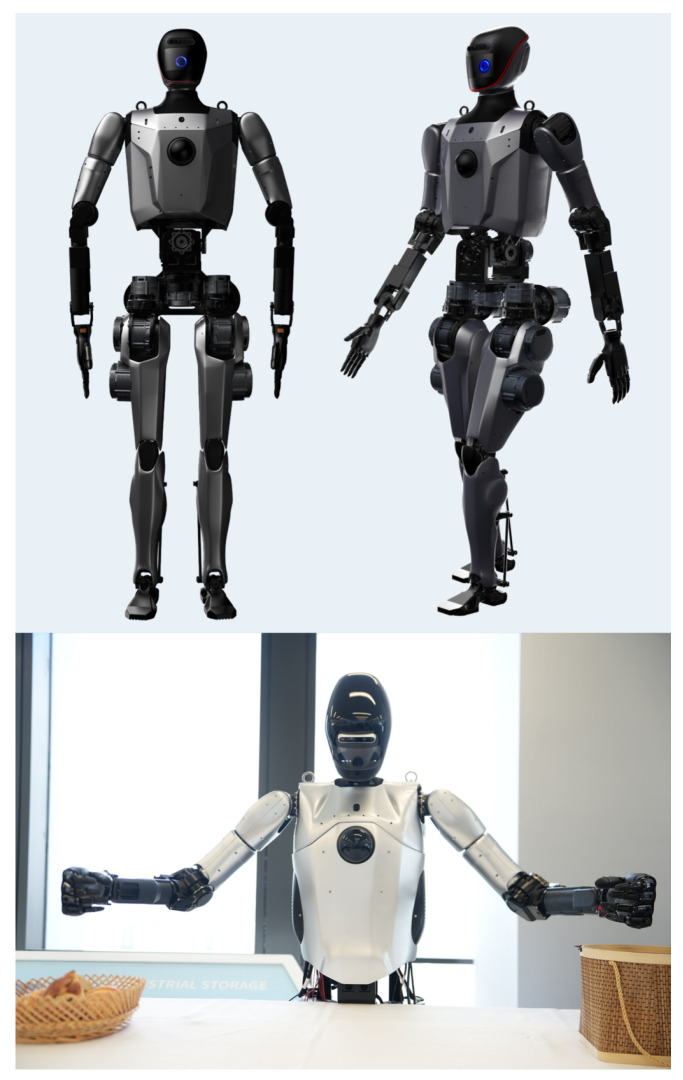
Full-size universal humanoid robot Loong.

This section systematically analyzes Loong’s design innovations through three critical dimensions. Figure 1 depicts the overall design and external appearance of the full-size universal humanoid robot, Loong. First, the highly biomimetic joint design enables Loong to exhibit anthropomorphic kinematic properties, particularly in the hip, waist, knee, and ankle articulations. Second, based on a multi-level control architecture incorporating efficient data exchange channels in kernel space and adaptive temporal control strategies, Loong significantly enhances the real-time performance and stability of the control system. Finally, employing a modular system integration strategy, Loong incorporates replaceable hardware modules including the head, mechanical arms, dexterous hands, and leg–foot assemblies, enabling adaptability across diverse application scenarios.

### 2.2. Lightweight Bionic Joints

**Figure 2:**
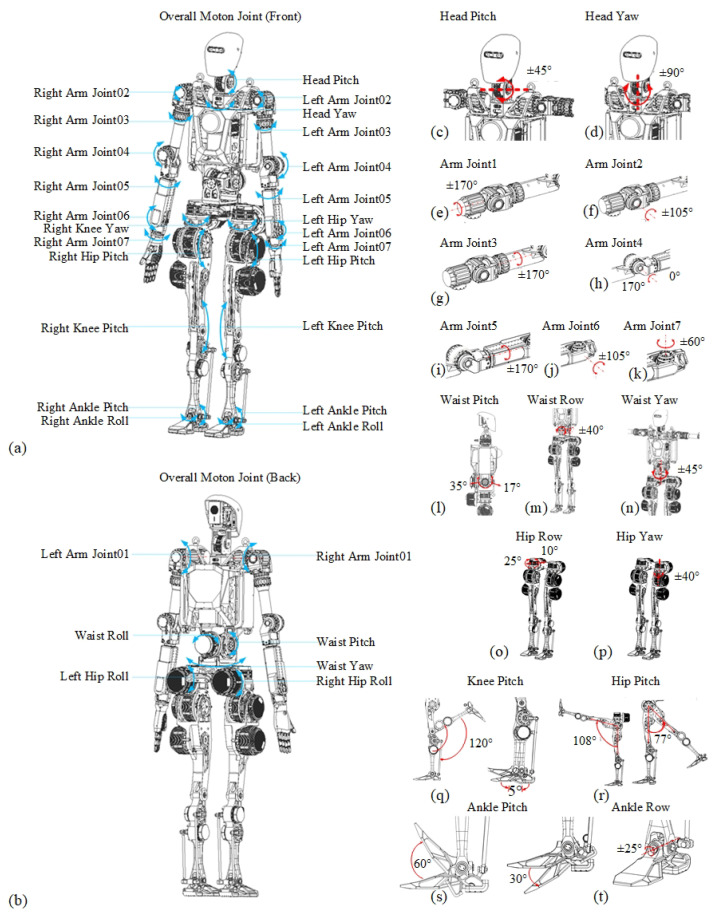
Overall motion joints of humanoid robot Loong and their motion range. The blue and red arrows denote the rotational direction of the motion range. (**a**) The overall motion joints in the front. (**b**) The overall motion joints on the back. (**c**,**d**) The pitch angle and yaw angle of the head. (**e**–**k**) The angle of the arm joints. (**l**–**n**) The pitch angle, row angle, and yaw angle of the waist. (**o**,**p**) The row and yaw angle of the hip. (**q**,**r**) The pitch angle of the knee and hip. (**s**,**t**) The pitch angle and row angle of the ankle.

The biomimetic approach adopted in Loong’s joint design is informed by recent studies on human biomechanics and robotic joint optimization [13]. Figure 2 presents the biomimetic joints design of Loong to replicate human motion while enhancing performance in dynamic environments. As synthesized by Hashimoto [14], traditional humanoid robots struggle to mimic human-like movements due to joint design limitations, and traditional serial or parallel joint configurations each present distinct trade-offs in terms of workspace, stiffness, and inertia. To overcome this, loong’s system constructs the overall design system of a human–machine fusion bionic mapping robot, and Loong’s design follows a systematic methodology, encompassing human anatomical mapping, biomimetic configuration [15], behavior topology clustering, and motion feature analysis [16]. This integrated approach enables the development of joints that accurately replicate the motion characteristics of human joints, focusing on the hip, waist, knee, and ankle regions [17]. Loong realized the development of highly dynamic, high-load-bearing, strong bionic, full-size humanoid robots.

The torso incorporates a three-degree-of-freedom waist assembly with a pitch motion that ranges from −17° to +35°, a roll motion range of ±40°, and yaw motion range of ±45°. The head mechanism features two degrees of freedom with a pitch motion range of ±45° and yaw motion range of ±90°.

The upper limb employs seven degrees of freedom per robotic arm. The shoulder has a pitch motion range of ±170°, a roll motion range of ±105°, and a yaw motion range of ±170°. The elbow mechanism delivers flexion motion range from 0° to 170° and rotational motion range of ±170°, while the wrist assembly enables pitch motion range of ±105° and roll motion range of ±60°.

The lower limb incorporates ankle joints with a pitch motion that ranges from −30° to +60° and a roll motion range of ±25°. Knee joints provide pitch articulation with motion that ranges from −120° to +5°, while the hip complex delivers three degrees of freedom: the pitch motion ranges from −77° to +105°, the roll motion ranges from −10° to +25°, and the yaw motion has a range of ±40°.

**Figure 3:**
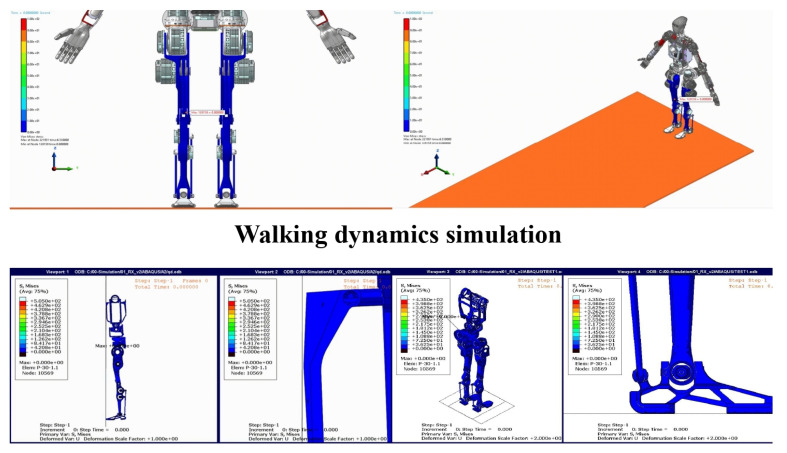
Simulations of walking dynamics, forward fall collision, and 0.5 m jump.

To ensure the reliability and stability of the humanoid robot prototype, a series of simulations were conducted, including walking dynamics, forward fall collision, and 0.5 m jump simulations. The results presented in Figure 3 demonstrated the robot’s ability to perform complex motions while maintaining stability and robustness. In the walking dynamics simulation, the robot exhibited stable bipedal locomotion, with proper weight distribution and balance control. The simulation confirmed that the robot could adapt to different walking speeds and surface conditions, showcasing its potential for real-world applications. The forward fall collision simulation tested the robot’s ability to recover from sudden disturbances. The results indicated that the robot could effectively absorb impact forces and regain stability, highlighting its robust design and control algorithms. Finally, the 0.5 m jump simulation validated the robot’s capability to perform dynamic motions. The robot successfully executed the jump, maintaining balance during takeoff and landing, which is critical for tasks requiring agility and precision. These results provide a strong foundation for further development and real-world deployment, demonstrating the robot’s potential for applications in complex environments.

**Figure 4:**
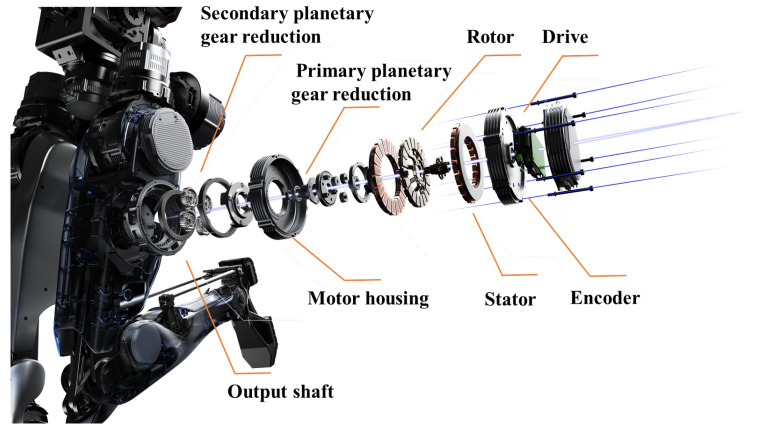
A high-performance integrated drive–sense–control joint module.

A high-performance integrated drive–sense–control joint module has been developed as presented in Figure 4, characterized by high density, high latitude, high speed, and high rigidity. The humanoid robot incorporates a total of 2 categories, 10 types, and 31 joints (excluding the dexterous humanoid hand). The maximum torque of the joints is 396 Nm, with a peak torque density of 200 Nm/kg [18]. The upper limbs utilize high-power-density motors coupled with high-precision harmonic drives, while the lower limbs employ axial flux motors combined with low-ratio planetary gear reducers.

Using a lightweight bionic joint design, we have resolved the bulkiness of the integrated servo actuator, achieving agile movement in complex environments [19]. These biomimetic joint designs are complemented by an innovative control scheme that integrates composite drive units and harmonic drive systems. In the lower limb joints, axial flux motors coupled with low-reduction ratio planetary gearboxes (QDD) deliver a peak torque density of 175 Nm/kg, ensuring stability during dynamic movement. For the upper limbs, a high-precision harmonic drive system (SEA) was employed to mimic the elastic transmission characteristics of human joints, enabling fine motion control through active compliance mechanisms. The joints are equipped with torque sensors and position feedback systems to ensure precise control over joint movements, closely mirroring human biomechanics.

### 2.3. Modular System Integration Strategy

Loong integrates multiple replaceable hardware modules, including the head, robotic arms, dexterous hands, and leg–foot assemblies, in order to meet the diverse functional requirements across various application scenarios [20]. These modules are designed with standardized mechanical and electrical interfaces for system integration.

The Loong head module integrates a depth camera, a microphone array, and a speaker, providing fundamental capabilities for target recognition and language interaction. When Loong is deployed in industrial manufacturing scenarios that require high precision, the head module can be easily replaced with one equipped with high-resolution cameras or LIDAR devices, enabling high-precision recognition and spatial perception.

**Figure 5:**
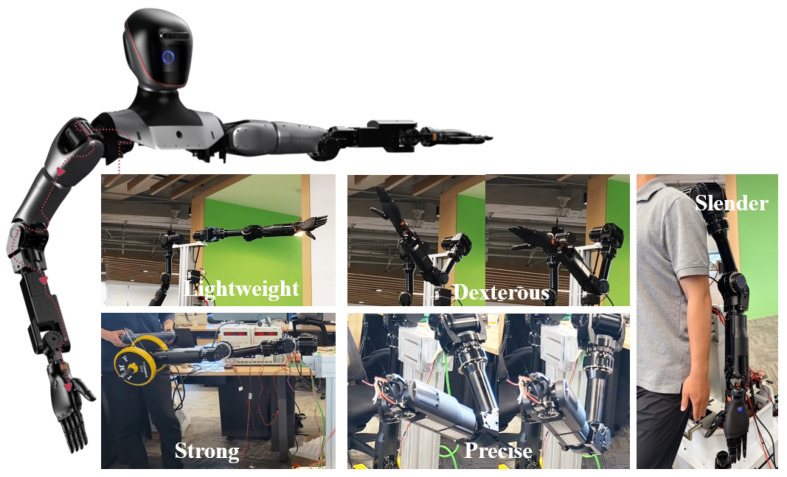
A lightweight robotic arm capable of dexterity, load, and precise operation.

The robotic arm of Loong, shown in Figure 5, possesses the following characteristics: Firstly, it is lightweight, with the entire arm weighing 5.6 kg. Secondly, it is dexterous, featuring 7 degrees of freedom and a working radius of 600 mm. Thirdly, it is strong, capable of a 4 kg payload at the end-effector. Fourthly, it is precise, with a repeat positioning accuracy of ±0.03 mm. Lastly, it is slender, with a large length-to-diameter ratio, allowing for greater freedom of movement.

**Figure 6:**
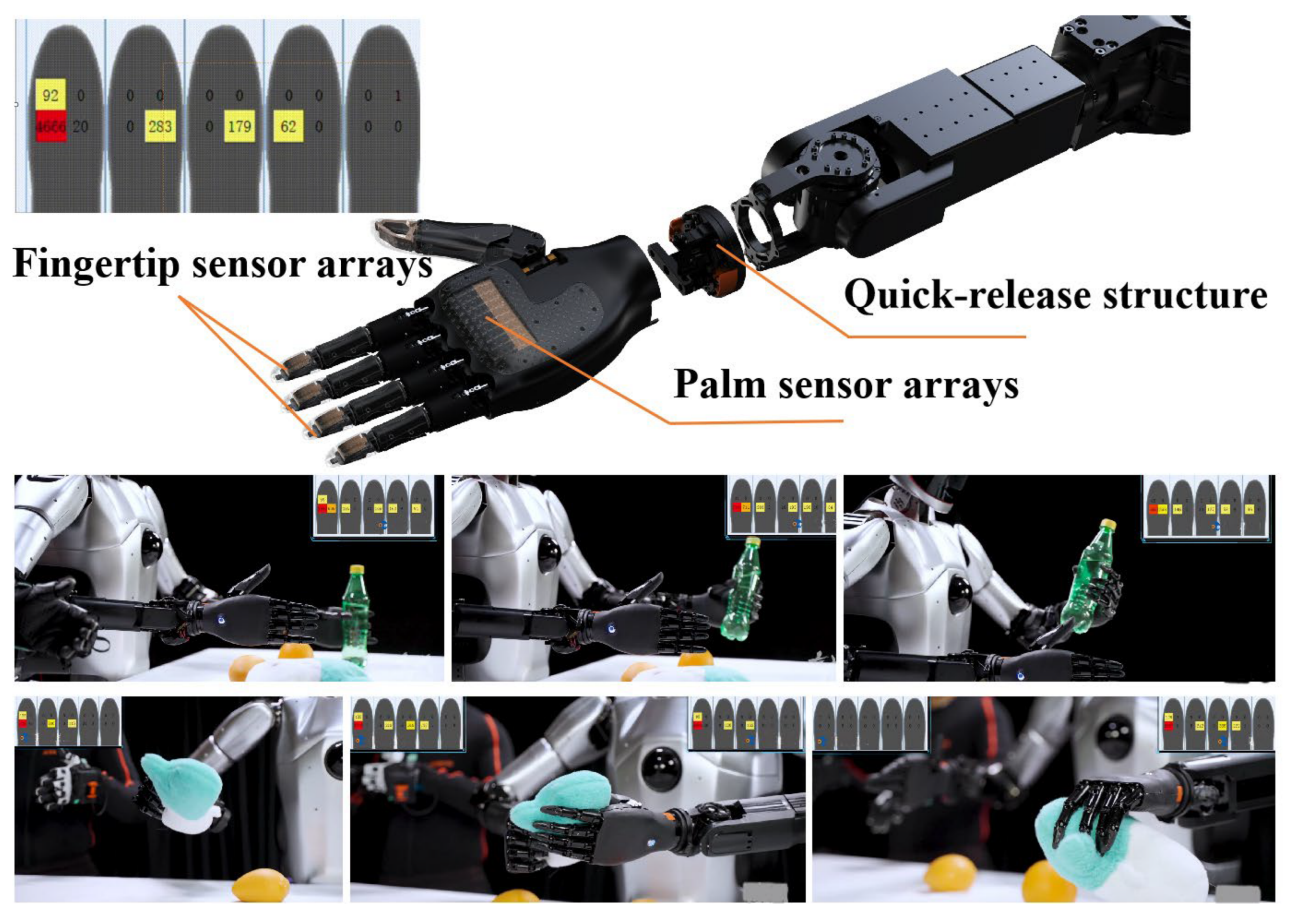
A dexterous hand with integrated fingertip sensor arrays and palm sensor arrays features a quick-release structure.

Based on the principle of biological inspiration, research into dexterous hands [21] aims to replicate the remarkable manipulation capabilities of the human hand. However, true dexterity stems not only from complex mechanical articulation but, more critically, from rich and precise perceptual feedback. Much like the human hand, which is densely innervated with tactile receptors across its skin, equipping a robotic end-effector with comprehensive tactile sensing—achieved through dense arrays of distributed sensors [22]—is fundamental to endowing it with a sense of touch. This tactile perception is the cornerstone for closing the loop in autonomous, intelligent interaction with unstructured environments. Following this bio-inspired design philosophy [23], we have developed a humanoid five-finger hand that integrates fingertip and palm sensor arrays. It features a quick-release structure for easy detachment from the robotic arm and possesses 19 degrees of freedom (6 active degrees of freedom). As shown in Figure 6, the entire hand weighs 550 g, with a single finger capable of exerting up to 15N of force, and the whole hand can handle a load of up to 5 kg. The fingers are equipped with a passive lateral swing structure, providing flexibility during interaction with objects, thereby enhancing the hand’s grasping capability.

Loong’s leg–foot assemblies module is designed for lightweight construction, high rigidity, and low inertia, which helps achieve high mobility and allows it to navigate through complex outdoor terrains. In flat-surface work environments, the operational efficiency of Loong can be significantly enhanced by configuring a mobile chassis.

The system-level modular design strategy in this study not only implements the efficiency of assembly and maintenance but also establishes a robust foundation for the robot’s rapid adaptation to diverse application scenarios. By leveraging the flexible combination and configuration of modules, Loong’s functional characteristics can be optimized for specific tasks, thereby significantly enhancing the practical value and application range of the robotic system.

### 2.4. Multi-Level Control Architecture

Compared to human capabilities, there is a noticeable gap in agility and autonomy in humanoid robots [24]. To address the complexities in controlling a full-sized humanoid robot, this study has designed a multi-level control architecture that facilitates efficient operation and high-performance execution, which separates fast proprioceptive stabilization from slow perceptual decision-making to achieve more robust locomotion [25,26]. Humanoid robots often face severe balance issues due to the heavy motion of their limbs. Hierarchical Multi-level Control Architecture can significantly alleviate this by reducing limb control errors [27].

**Figure 7:**
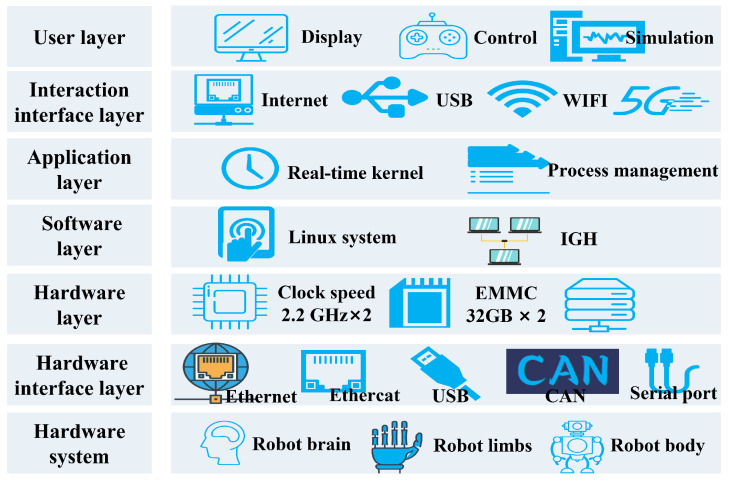
Intelligent hierarchical control system architecture.

Figure 7 presents the intelligent hierarchical control system architecture of Loong. It incorporates a dual-core heterogeneous computing architecture, integrating two 2.2 GHz CPUs and two 930 MHz GPU coprocessors, which provides a total computing power of 400TOPS. The CPUs handle real-time control tasks, while the GPU coprocessors execute parallel processing for computationally intensive operations, such as perception algorithms. This allocation strategy of computing resources ensures the timely execution of high-level control commands while supporting the complex processing demands of lower-level control functions.

**Figure 8:**
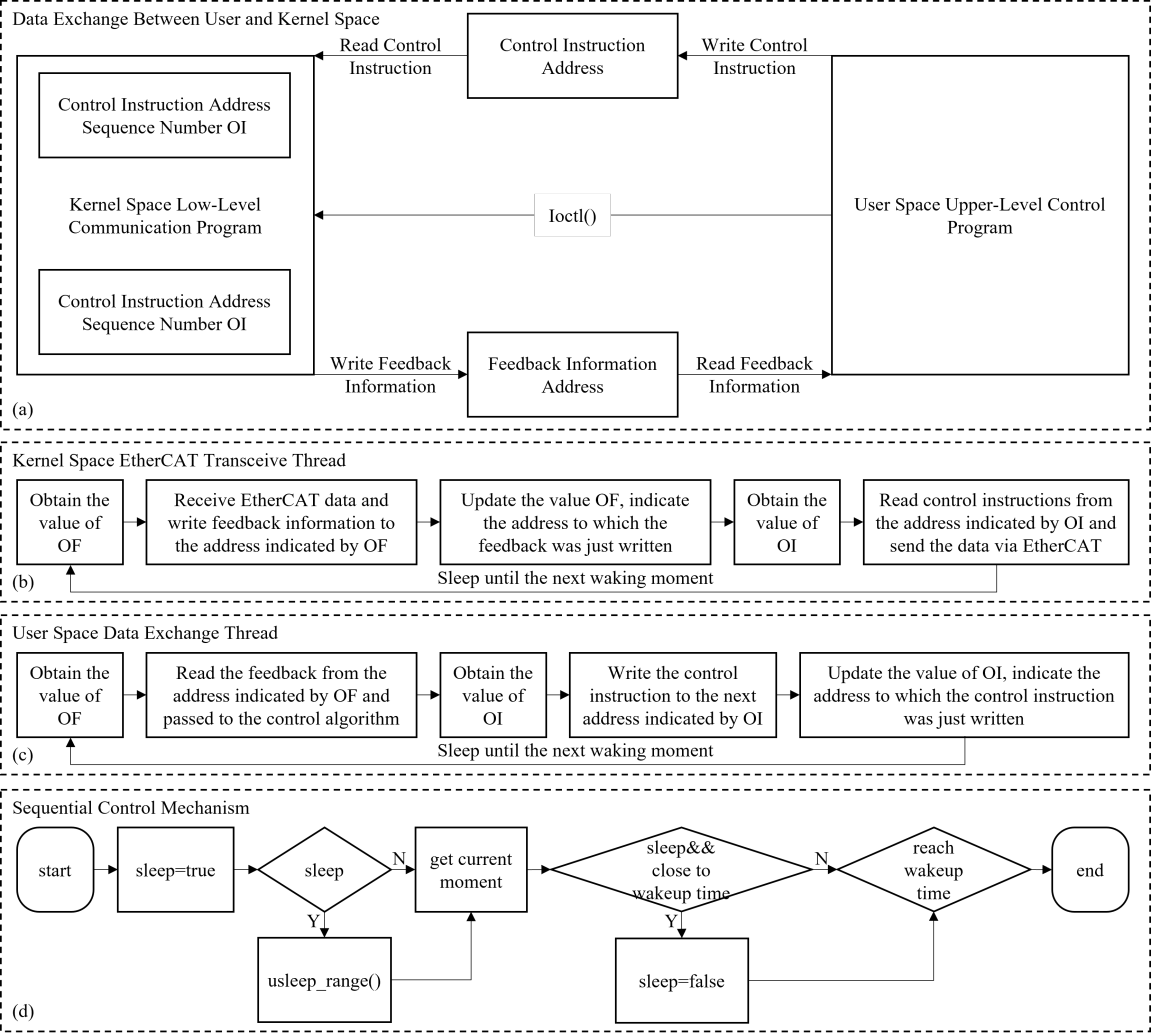
Multi-level control architecture. (**a**) The data exchange between user and kernel space. (**b**) The kernel space EtherCAT transceive thread. (**c**) The user space data exchange thread. (**d**) The sequential control mechanism.

We designed a multi-level control architecture, as detailed in Figure 8. It builds a efficient data exchange channel between user space and kernel space by statically allocating six distinct memory segments within the kernel space: three for control instructions and three for feedback information. OI (Ordinal Instruction) and OF (Ordinal Feedback) are two critical state variables in this system to manage the data flow. Specifically, OI indicates the read/write address of control instructions, while OF indicates the read/write address of feedback information. This architecture enables the efficient transmission of control instructions and feedback information across different memory spaces and maintains the data consistency and real-time performance.

In addition, to address the efficiency of data exchange between real-time control tasks, we have modified the Read-Copy Update (RCU) mechanism concept widely adopted in the Linux kernel to enhance real-time performance [28], mitigating the performance overhead typically associated with conventional locking mechanisms while maintaining data consistency. In this implementation, writing thread writes new data to the next available address and, upon completing the writing operation, updates the corresponding state variables (OI or OF). Reading thread subsequently retrieves the latest data based on the address specified by the state variables.

Finally, to ensure real-time control, we raise an innovative approach by combining the usleep_range() mechanism with spin-waiting, developing an adaptive timing control strategy. When the time to the next control cycle is more than 12% of the control period, the usleep_range() mechanism is used for inducing sleep to minimize system resource consumption. On the contrary, as the time to the next control cycle is less than the 12% of the control period, the system switches to a spin-waiting mode to ensure precise sequential control. This dynamic switching strategy can maintain the control cycle jitter within a range of ±20 microseconds, which effectively balances the real-time performance with resource utilization of the system.

**Figure 9:**
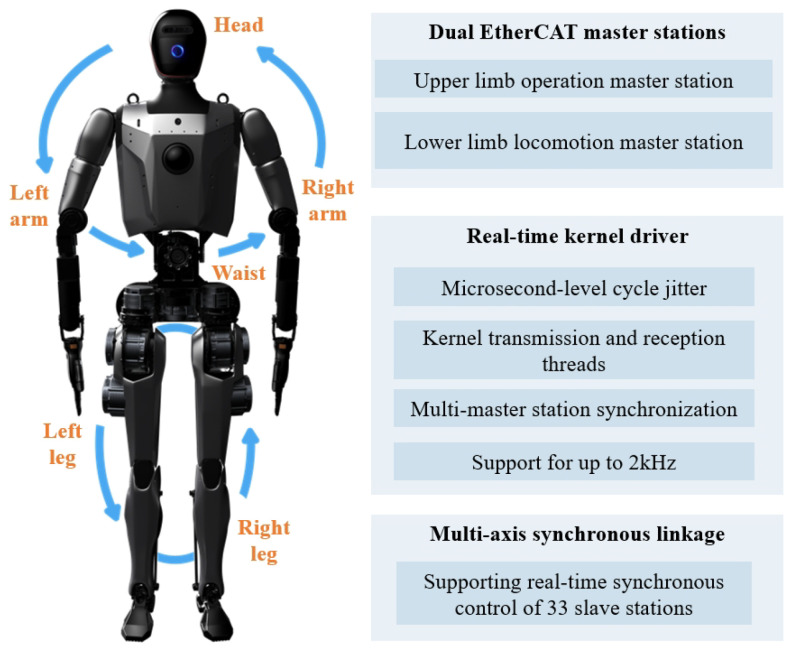
A multi-master real-time synchronous high-speed EC bus system.

To enable efficient communication, as shown in Figure 9, Loong adopts a dual EtherCAT master configuration, where each master station is respectively responsible for controlling upper limb operations and lower limb walking. Each master station is connected to its associated slaves through a high-speed, real-time bus [29]. This distributed communication framework enables high-frequency control of 33 slave stations at 2 kHz, significantly enhancing the system’s real-time control capabilities.

## 3. Experiments and Results

This section presents the experimental validation of Loong, which is divided into basic performance testing and motion scenario testing. The former involves collecting data from each joint of the robot while it marches in place for basic performance analysis, while the latter tests the robot’s mobility in simple scenarios.

### 3.1. Basic Performance Testing

As shown in Table 1, this robotic platform demonstrates a series of robust locomotion performance parameters. The test results indicate that its maximum moving speeds reach 1 m/s in walking mode and 2 m/s in running mode, while maintaining a load capacity of 10 kg under stable gait conditions. In terms of environmental adaptability, the robot not only operates effectively on various terrains including cement, gravel, and sandy soil but also maintains balance under an external thrust of 50 N and can stably traverse slopes up to 10°. Its continuous operation time per charge reaches approximately 40 min, with safety ensured by a dual-mode emergency stop system incorporating both onboard and remote activation.

**Figure 10:**
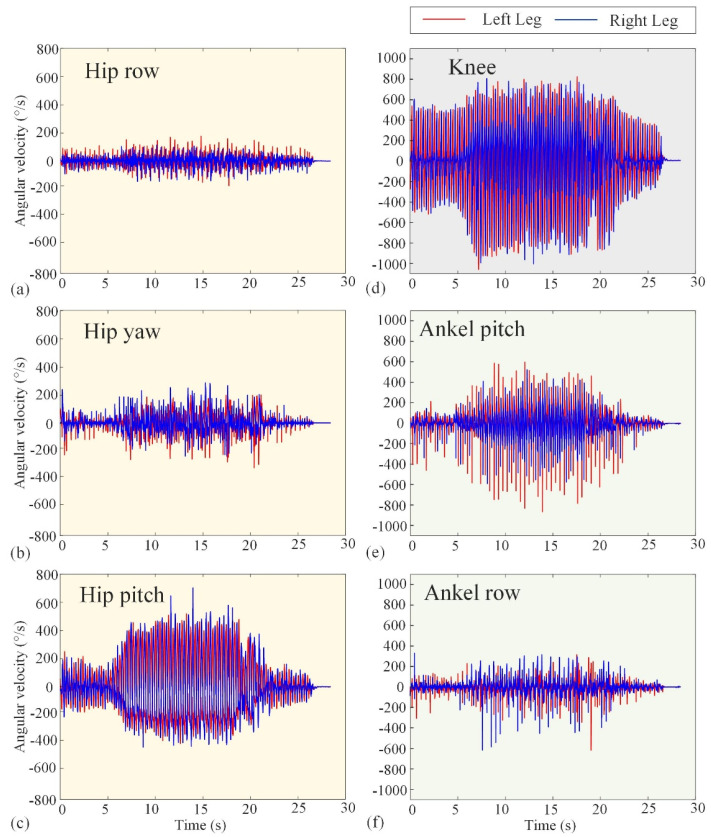
Experimental curves of angular velocity of the hip, knee, and ankle joints. The red lines represent the left leg, while the blue lines represent the right leg. (**a**–**c**) The row angular velocity, yaw angular velocity, and pitch angular velocity of the hip joint, respectively. (**d**) The knee angular velocity. (**e**,**f**) The pitch angular velocity and row angular velocity of the ankle joint, respectively.

**Figure 11:**
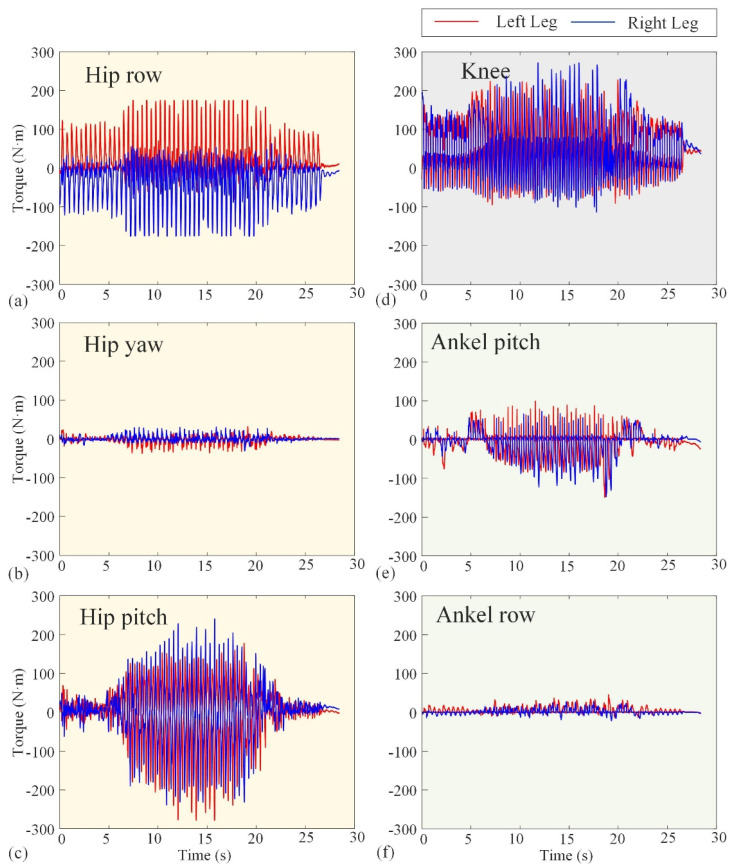
Experimental curves of torque of the hip, knee, and ankle joints. The red lines represent the left leg, while the blue lines represent the right leg. (**a**–**c**) The row torque, yaw torque, and pitch torque of the hip joint, respectively. (**d**) The knee torque. (**e**,**f**) The pitch torque and row torque of the ankle joint, respectively.

**Figure 12:**
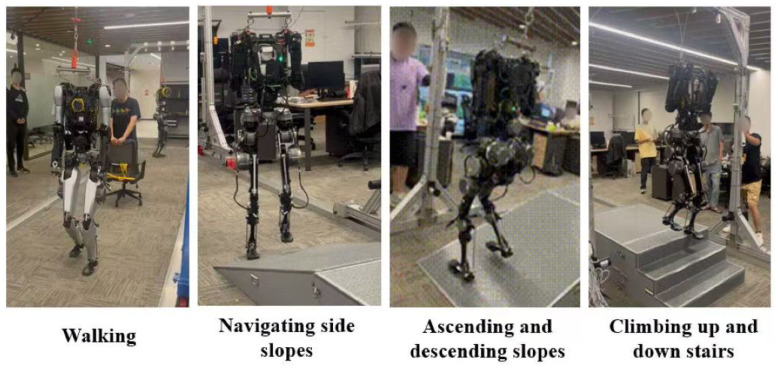
The humanoid robot prototype underwent functional verification tests.

The basic performance tests conducted in Section 3.1 validate the reliability of Loong’s hardware system and the effectiveness of its underlying control strategies. Figure 10 and Figure 11 represent the experimental angular velocity and torque, respectively. The angular velocities at several (hip, knee, and ankle) joints are measured by the encoders attached to the velocity–displacement actuators at their respective positions, while the torques are derived from the current loop. The angular velocities in Figure 10 indicate that the actuators will not operate at more than 180 rpm. Also, the maximum operating torque can be seen in Figure 12 as not exceeding 300 Nm. These results, combined with the comprehensive performance metrics summarized in Table 1, confirm that the robotic platform operates stably and robustly under its specified working conditions, providing a solid foundation for the motion scenario tests that follow.

### 3.2. Motion Scenario Testing

Figure 12 presents the humanoid robot prototype that underwent functional verification tests, including walking, ascending and descending slopes, navigating side slopes, and climbing up and down stairs. These tests confirmed the robot’s ability to handle various terrains and obstacles, showcasing its potential for real-world applications in environments requiring complex mobility. The results indicate satisfactory performance in terms of balance control, adaptability, and energy efficiency during locomotion tasks.

**Figure 13:**
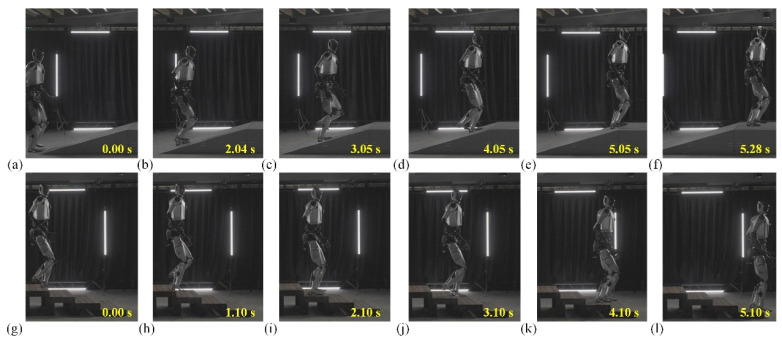
Validation of motion scenarios for humanoid robot Loong going uphill and down stairs. (**a**–**f**) Snapshots of Loong going uphill in 5.28 s. (**g**–**l**) Snapshots of Loong going down stairs in 5.10 s. The height and inclination of the slope are 600 mm and 20°, respectively, and there are three steps in total; each step is 150 mm high.

Figure 13 consists of eight frames, with two distinct sets of images: one depicting the robot going uphill, and the other showing it descending stairs. In the first row, the robot begins its ascent (Figure 13a–f) at 0.00 s, where its left foot makes contact with the foot of the slope. The robot’s posture appears upright, and it maintains a steady, balanced position. As time progresses, we can observe the robot’s fluid movement, with the right leg preparing to follow, lifting toward the next step, while the left leg starts to push against the surface for the next step. In the second row, starting from frame g at 0.00 s, the robot begins its descent. The action is reversed in comparison to the first row, as the right foot steps down, followed by the left. The robot’s stance is slightly different here: it appears more cautious, balancing its weight to avoid falling, with its knees bent and body slightly lowered to ensure stability. The robot’s use of arm movement in this action could be inferred from its dynamic posture as it adapts to the different motions required for climbing versus descending.

Throughout the sequence, the robot uses synchronized movements of the legs and arms, resembling a human-like gait, with small adjustments made in the positioning of the upper body for better balance. Figure 13 also shows a steady progression, where each movement builds naturally upon the last, allowing for the robot to adapt to the irregularity of the steps, such as the height difference in each frame. The time stamps across the frames (0.00 s to 5.28 s in robot going uphill and 0.00 s to 5.10 s in robot going down stairs) suggest that the robot’s movement is relatively smooth and efficient, with no sudden jerks or awkward pauses. Stable robot traversal over steps and slopes is achieved through compliant ankle and hip control, where real-time stiffness and damping adjustments compensate for terrain-induced disturbances. This indicates that the robot’s design is highly advanced, capable of performing complex tasks with high precision and fluidity.

**Figure 14:**
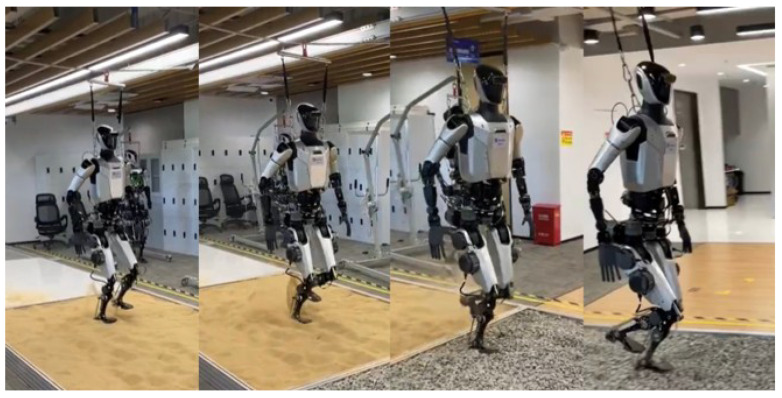
Adaptive locomotion on varied terrains.

Figure 14 presents an experimental demonstration of our full-size humanoid robot, Loong, autonomously traversing unstructured outdoor terrains. As shown, Loong successfully adapts its bipedal locomotion to challenging surfaces, including loose sand and gravel. The robot dynamically adjusts its foot placement, step timing, and upper-body posture to maintain balance and prevent sinking or slipping. This experiment validates the robustness and generalizability of our whole-body control framework for humanoid robots in real-world, uncertain environments.

**Figure 15:**
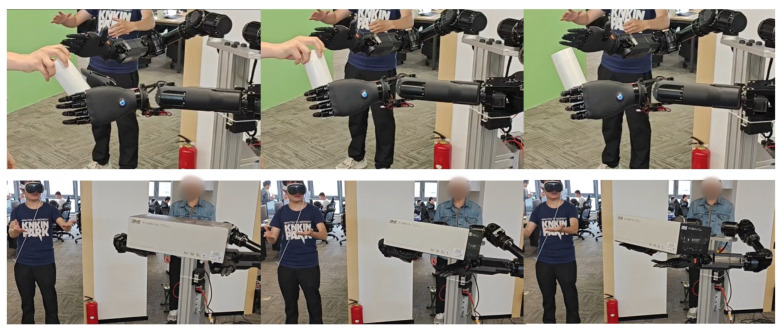
Human-in-the-loop.

**Figure 16:**
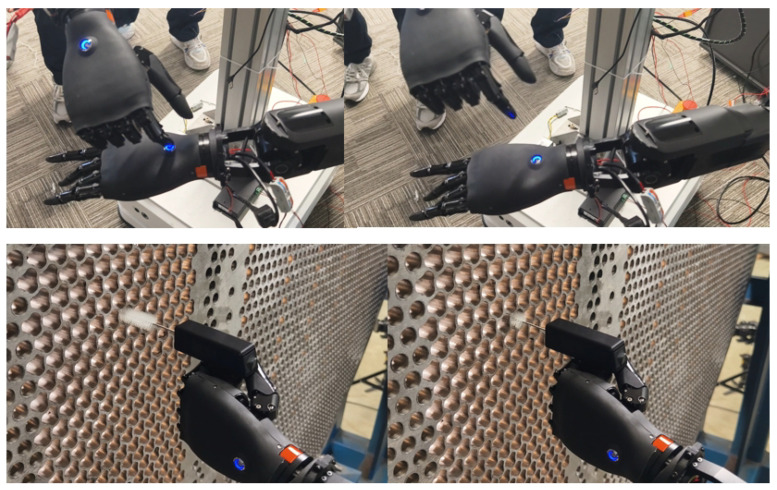
Fine manipulation.

**Figure 17:**
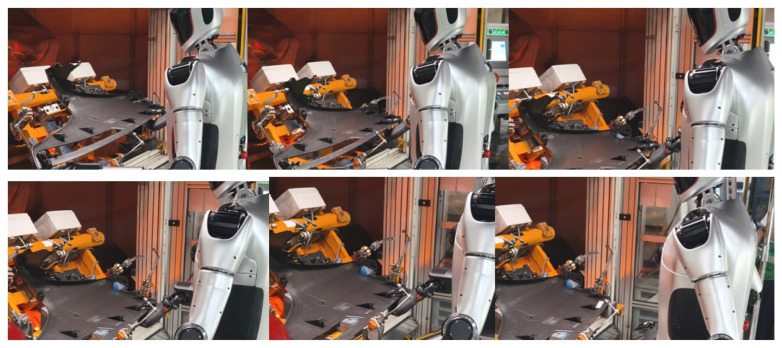
Loading and unloading of automotive parts.

As presented in Figure 15, our research team continuously trained and optimized the operation model of Loong through a human-in-the-loop framework, thereby enhancing its accuracy and reliability [30]. This iterative process facilitates the execution of operation tasks, such as identifying the switch position and performing the triggering action to activate it, as well as executing precise cleaning operations on heat sinks, as shown in Figure 16. Furthermore, as demonstrated in Figure 17, Loong is capable of efficiently handling workpieces on industrial manufacturing production lines, including tasks such as loading and unloading components.

## 4. Conclusions

This paper has presented the development of Loong, an open-source full-size universal humanoid robot platform designed to address key challenges in practical full-sized humanoid robotics. Using a systematic approach, including biomimetic joint design, multi-level control architecture, and modular system integration, we have successfully developed a humanoid robot Loong with enhanced capabilities for real-world applications.

Future work includes comprehensive field testing of Loong in various real-world environments to validate its performance under different conditions. We plan to conduct experiments in industrial manufacturing settings, which will provide valuable feedback for further refinements.

We also intend to expand the open-source community around Loong, encouraging the collaborative development of new modules and control algorithms that can further extend its capabilities. Our ultimate goal is to advance Loong toward practical applications in real-world scenarios, creating viable markets for full-size humanoid robots in industrial manufacturing, public service, and specialized sectors. By sharing this platform as an open-source resource, we aim to accelerate innovation in humanoid robotics and promote standardization across the field, contributing to the broader adoption of humanoid robots for practical use.

## Figures and Tables

**Table 1:** Capability Parameters.

Capability Item	Parameter/Status	Description
Maximum Moving Speed	Walking: 1 m/s	Safety speed limit (verified in indoor environments)
	Running: 2 m/s	
Load Capacity	10 kg	Maximum load capacity under stable walking conditions
Terrain Adaptability	Pass	Adaptable to cement/road/gravel/sandy soil surfaces, ground fluctuation tolerance ≥ 5 cm
Anti-interference Ability	50 N thrust	Maintains stability during rhythmic gait walking
Slope Traversal Capability	10° slope	Able to ascend and descend slopes forward under no-load conditions
Continuous Walking Time	40 min	Stable walking at room temperature 23 °C until motor temperature rise reaches 60 °C
Emergency Stop System	Dual-mode pass	On-board emergency stop + remote emergency stop

## Data Availability

The datasets and related open-source resources used in this study are available through the OpenLoong Open Source Community.
